# Tuberculosis Diagnostics and Localization in Chest X-Rays via Deep Learning Models

**DOI:** 10.3389/frai.2020.583427

**Published:** 2020-10-05

**Authors:** Ruihua Guo, Kalpdrum Passi, Chakresh Kumar Jain

**Affiliations:** ^1^Department of Mathematics and Computer Science, Laurentian University, Greater Sudbury, ON, Canada; ^2^Department of Biotechnology, Jaypee Institute of Information Technology, Noida, India

**Keywords:** tuberculosis, chest X-ray, manifestations, localization, convolutional neural networks, artificial bee colony algorithm, ensemble, class activation mapping

## Abstract

For decades, tuberculosis (TB), a potentially serious infectious lung disease, continues to be a leading cause of worldwide death. Proven to be conveniently efficient and cost-effective, chest X-ray (CXR) has become the preliminary medical imaging tool for detecting TB. Arguably, the quality of TB diagnosis will improve vastly with automated CXRs for TB detection and the localization of suspected areas, which may manifest TB. The current line of research aims to develop an efficient computer-aided detection system that will support doctors (and radiologists) to become well-informed when making TB diagnosis from patients' CXRs. Here, an integrated process to improve TB diagnostics via convolutional neural networks (CNNs) and localization in CXRs via deep-learning models is proposed. Three key steps in the TB diagnostics process include (a) modifying CNN model structures, (b) model fine-tuning via artificial bee colony algorithm, and (c) the implementation of linear average–based ensemble method. Comparisons of the overall performance are made across all three steps among the experimented deep CNN models on two publicly available CXR datasets, namely, the Shenzhen Hospital CXR dataset and the National Institutes of Health CXR dataset. Validated performance includes detecting CXR abnormalities and differentiating among seven TB-related manifestations (consolidation, effusion, fibrosis, infiltration, mass, nodule, and pleural thickening). Importantly, class activation mapping is employed to inform a visual interpretation of the diagnostic result by localizing the detected lung abnormality manifestation on CXR. Compared to the state-of-the-art, the resulting approach showcases an outstanding performance both in the lung abnormality detection and the specific TB-related manifestation diagnosis vis-à-vis the localization in CXRs.

## Introduction

Tuberculosis (TB), a highly contagious lung disease, is the leading cause of worldwide death followed by malaria and HIV/AIDS. The World Health Organization (World Health Organization, 2018) alludes that more than 95% of TB patients live in developing countries that lack adequate healthcare funding and supporting medical infrastructure. In descending order, two-thirds or 67% of newly TB-infected cases occur in eight developing nations beginning with India, followed by China, Indonesia, the Philippines, Pakistan, Nigeria, Bangladesh (formerly, East Bengal of British India), and South Africa. Statistics from 2000 to 2018 have projected a saving of 58 million lives via early TB diagnosis and timely treatment. Thus, timeliness in TB diagnosis is critical when mitigating its spread, improving TB preventive efforts and/or minimizing the TB death rate.

Currently, computed tomography (CT) offers the best-known TB detection method. For most earlier cases, however, TB diagnosis is confirmed via chest X-rays (CXRs) given the radiation dose, cost, availability, and the ability to reveal the unsuspected pathologic alterations among TB detection methods. For decades, researchers have focused on developing a computer-aided detection (CAD) system for the preliminary diagnosis of TB-related diseases via medical imaging. In the early stages, CAD depends on rule-based algorithms to select and extract useful pathogenic features within images to yield meaningful quantitative insight; yet, such methods are time-consuming, having to rely chiefly on the artificial extraction of patterns with useful information. As the manifestation of many diseases typically covers an extremely small region of the entire image, the challenge of the feature recognition process quickly becomes compounded. Moreover, with cumulative medical image data and evolving mutations of the disease, problems such as poor transferability among different datasets and unstable performance vis-à-vis newly generated data have stopped the CAD system from formulating a well-grounded decision with high accuracy.

With advances in deep learning, the convolutional neural networks (CNNs) have consistently surpassed other traditional recognition algorithms in achieving superordinate performance for image-based classification and recognition problems. The superlative ability to automatically extract useful features from the inherent characteristics of data makes CNN the first choice for complex medical problem solving. To date, CAD systems embedded with deep-learning algorithms have worked efficiently for medical disease detection by effectively generating a range of high-quality diagnostic solutions while spotlighting suspicious features.

## Related Work

Historically, the CAD system for disease diagnosis relies mainly on feature extraction and pattern recognition technology. Khuzi et al. (2009) employed the gray-level co-occurrence matrix, a texture descriptor via the spatial relationship between different pixel pairs, to identify masses from mammograms. In Yang et al. (2013), presented a successful application of gray-scale invariant features to detect tumor from breast ultrasound images. Jaeger et al. (2014) proposed an automatic TB detection system by computing texture and shape features from CXRs using local binary pattern. The extracted features are then fed into a binary classifier to produce normality-pathology diagnosis with respective resulting accuracies of 78.3% and 80% on two smaller datasets, namely (a) the local health department privately permitted use of one dataset and (b) the publicly available Shenzhen Hospital CXR dataset.

Lately, with the rising popularity of deep-learning models coupled with the superb CNN performance within the computer vision field, much research applying CNN models in disease diagnosis via medical imaging has emerged. Anthimopoulos et al. (2016), for example, introduced a deep CNN model with dense structure and applied it to diagnose interstitial lung diseases via CT images, generating a higher-density resolution view of lung parts in either two-/three-dimensional formats compared to CXRs. Interestingly, six different lung disease manifestations together with healthy cases may be distinguished via their proposed model with a demonstrable accuracy of >80%. Still, as the dataset used in the study of Anthimopoulos et al. was limited to only 120 CT images, the transferability and the generalizability of their proposed model must now be further validated. In Lakhani and Sundaram (2017), two deep CNN models, AlexNet and GoogLeNet, were applied by Lakhani et al. to classify the chest radiographs as pulmonary TB vs. healthy cases vis-à-vis the Shenzhen Hospital CXR dataset and the Montgomery County CXR dataset. Here, the complete datasets were apportioned into training (68.0%), validation (17.1%), and testing (14.9%) sets, respectively. Areas under the curve (AUCs) were used for statistical analysis of overall performance; in their reports, the researchers noted achieving the best classifier with AUC of 0.99.

More recently, Pasa et al. (2019) presented an automated diagnosis with the localization of TB on the same two datasets vis-à-vis a deep CNN with the shortcut connection. Although not as good as the previously reported work in that the best AUC achieved here is 0.925, the localization result generated using saliency maps is, nevertheless, quite impressive. Notwithstanding, while the CNN models all achieved satisfying results on the detection of pulmonary TB in the previous two experiments, the performance test on the more complicated but practical task of diagnosing among multiple lung diseases remains elusive. In Rajpurkar et al. (2018), proposed a 121-layer dense CNN architecture and tested the model by training with the currently largest publicly available chest radiography dataset, the National Institutes of Health (NIH) CXR dataset, to detect more than 10 different lung diseases. The performance achieved by the CNN model has then been compared to that performed by radiologists; accordingly, the proposed model achieves an F1 score of 0.435, which exceeds the average performance given by human experts of >0.387. Even so, without knowing the classification accuracy for each disease, the robustness of the result of Rajpurkar et al. (2018) cannot yet be ascertained. Rajaraman and Antani (2020) recently proposed a modality-specific deep-learning model that evaluates the efficacy of knowledge transfer gained through an ensemble of modality-specific deep-learning models toward improving the state-of-the-art in TB detection.

Lopez-Garnier et al. (2019) trained and evaluated a CNN for automatic interpretation of Microscopic Observed Drug Susceptibility (MODS) cultures digital images. The MODS is a test to diagnose TB infection and drug susceptibility directly from a sputum sample in 7–10 days with a low cost and high sensitivity and specificity, based on the visual recognition of specific growth cording patterns of *Mycobacterium tuberculosis* in a broth culture. Despite its advantages, MODS is still limited in remote, low-resource settings, because it requires permanent and trained technical staff for the image-based diagnostics. Hence, it is important to develop alternative solutions, based on reliable automated analysis and interpretation of MODS cultures.

Liu et al. (2017) proposed a novel method using CNN to deal with unbalanced, less-category X-ray images. Their method improves the accuracy for classifying multiple TB manifestations by a large margin. They explored the effectiveness and efficiency of shuffle sampling with cross-validation in training the network and find its outstanding effect in medical images classification. They achieved an 85.68% classification accuracy in a large TB image dataset from Peru, surpassing any state-of-art classification accuracy in this area. Their methods and results show a promising path for more accurate and faster TB diagnosis in healthcare facilities in low- and middle-income countries.

Norval et al. (2019) investigated the accuracy of two methods to detect pulmonary TB based on the patient CXR images using CNNs. Various image preprocessing methods were tested to find the combination that yields the highest accuracy. A hybrid approach using the original statistical CAD method combined with neural networks was also investigated. Simulations were carried out based on 406 normal images and 394 abnormal images. The simulations showed that a cropped region of interest coupled with contrast enhancement yielded excellent results. When images were further enhanced with the hybrid method, even better results were achieved. They used Shenzhen Hospital X-ray set and Montgomery County X-ray set.

## Materials and Methods

### Datasets and Preprocessing

Two public CXR datasets, the Shenzhen Hospital CXR dataset and the NIH CXR dataset, have been used in our study to test the performance of deep CNN models processed via our proposed methodology.

The Shenzhen Hospital CXR dataset (Candemir et al., 2013; Jaeger et al., 2014; Wang et al., 2017) is compiled by the Shenzhen No. 3 People's Hospital and Guangdong Medical College in China. The dataset comprises 662 frontal posteroanterior CXR images in various sizes, among which 326 have been diagnosed as normal cases, whereas the other 336 as having TB manifestations. The NIH CXR dataset (Shin et al., 2016b) is by far one of the largest public CXR datasets. This dataset is extracted from the clinical PACS database at the NIH Clinical Center, comprising 112,120 frontal view (posteroanterior and anteroposterior) CXR images with 14 thoracic pathologies (atelectasis, consolidation, infiltration, pneumothorax, edema, emphysema, fibrosis, effusion, pneumonia, pleural thickening, cardiomegaly, nodule, mass, and hernia). As it is not anticipated for the original radiology report to be shared publicly, the disease information and labels for CXRs had to be text-mined via natural language processing techniques with accuracy of >90%. Owing to the massive amount of data, detailed annotations, and wide range of thorax diseases covered by this dataset, many researchers studying thorax disease detection vis-à-vis the deep-learning area have used it.

To improve the general quality of the NIH CXR dataset, all CXR images have been enhanced using contrast limited adaptive histogram equalization (CLAHE) (Pizer et al., 1987) with the clip limit number equals 1.25; similarly, to improve the processing speed, all CXR images have also been resized from their original size to 512 × 512. Also, in diagnosing TB-related manifestations on the NIH CXR dataset, as the distribution of CXRs under each TB-related disease class presents a strongly biased trend, models trained on this dataset will tend to perform with a strong preference for their conforming predictions. As such, data augmentation techniques such as horizontal flip, rotate, contrast adjustment, and position translation have been perspicaciously implemented to increase the number of images under the classes with fewer CXRs, thereby propagating an evenly distributed data to eliminate the interference.

### Methodology

**Figure 1:**
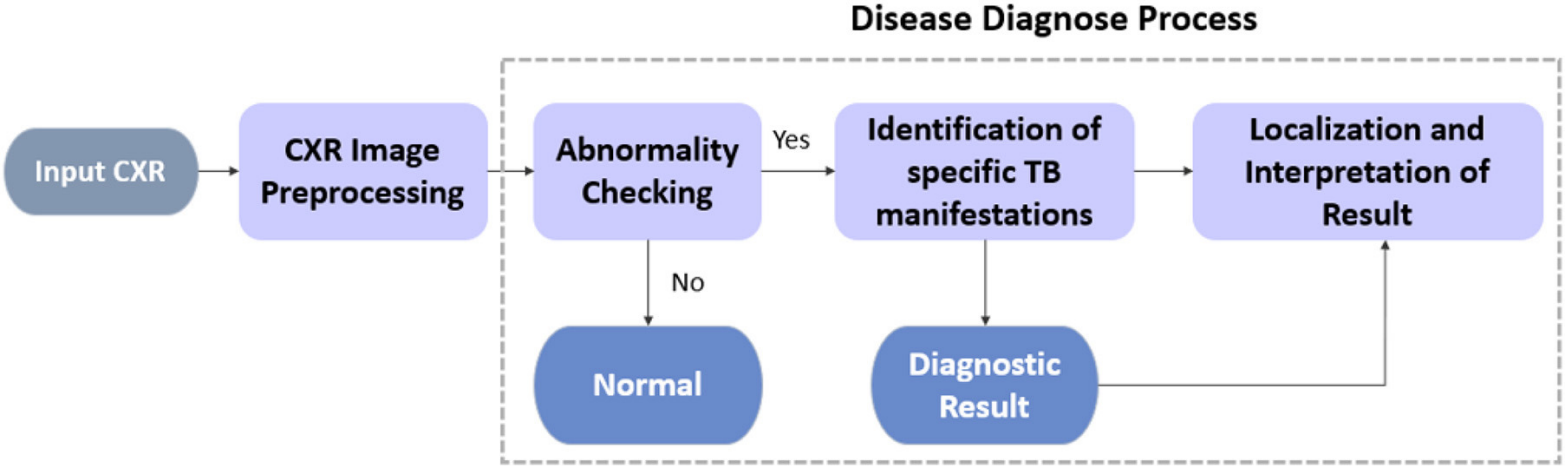
TB diagnosis pipeline.

In this section, a CAD system driven by deep CNN models for TB diagnostics and localization from CXR images with the use of unified approaches to improve the accuracy-stability of the disease detection process is proposed. As shown in Figure 1, we divide the TB diagnostic task into four subprocesses: (i) CXR image preprocessing; (ii) preliminary detection of the suspected TB patients via abnormality checking; (iii) identification of the specific TB manifestation [consolidation (Adler and Richards, 1953), effusion (Vorster et al., 2015), fibrosis (Chung et al., 2004), infiltration (Mishin et al., 2006), mass (Cherian et al., 1998), nodule (Kant et al., 2007), and pleural thickening (Gil et al., 1994)]; and (iv) localization of the suspicious diseased area on CXRs.

For each process, all deep CNN models used herein have been improved by structural modification, the implementation of artificial bee colony (ABC) algorithm during fine-tuning, and the amalgamation of an ensemble model. Unlike standard object detection task encapsulating bounding box information, the localization of TB manifestations is achieved primarily via class activation mapping; essentially, this entails the production of an attention map over the image to spotlight all the detected suspicious areas instead of predicting edges of bounding boxes.

Specifically, we analyze the overall performance of the experimented deep CNN models on two publicly available CXR datasets, namely, the Shenzhen Hospital (CXR) Dataset and the NIH CXR dataset. For all datasets, our analysis emphasizes the binary classification of CXR images to differentiate among the TB abnormalities, while performing a further diagnosis and localization of specific TB-related manifestation on the NIH CXR dataset.

Compared to typical performance results from just a plain application of the original deep CNN models, our quantitative results of the proposed methodology achieve a staggering improvement of >30% points during the identification of TB manifestation, aside from outstanding prediction accuracy with an average improvement of over >8% points on the abnormality detection. For qualitative results, not only can our model provide the attention map that fully encapsulates the suspicious diseased regions vis-à-vis the diagnostic results, but the model also can successfully distinguish among diseases caused by similar reason(s). Additionally, for spatially spread out TB-related manifestations, regardless of their sizes, the model can often localize the abnormalities successfully.

### Models and Architecture

#### CNN-Based Classification Model

CNN, a discriminative classifier developed from multilayer perceptron, is designed to recognize specific patterns directly from image pixels with minimal preprocessing. Owing to its hierarchical structure in propagating shift-invariant classification, CNN is also known as shift-invariant artificial neural networks (Zhang et al., 1988). Its unfailing ability to extract global features and contextualize information from the inherent characteristics of data makes CNN among the first choice for handling those most challenging scenarios.

Traditional CAD systems using machine-learning methods such as support vector machines and other techniques (e.g., K-nearest neighbors) have aided radiologists to improve their diagnostic accuracy; however, many of these earlier methods need to extract disease features manually. Moreover, the evolving nature and multifaceted features of lesions make features extracted in previous studies non-trivial when trying to reapply them to new patient data. Accordingly, traditional machine-learning methods are not suited for long-term effective solutions. Nowadays, the workload of radiologists has increased significantly alongside new advances in the radiology medical equipment and technology, big data diagnostics, and massive number of medical images being generated daily. In this sense, instead of traditional methods, the application of CNN in various diagnostic modalities appears to be both effective and efficient because of its ability to automatically extract from the image data complex pathological features while satisfying the intrinsic requirement of massive volumes of data.

In exploring the multiple and more popular deep CNN models for TB diagnosis and localization, we examined VGGNet (Boureau et al., 2011), GoogLeNet Inception Model (Szegedy et al., 2015), and ResNet (He et al., 2016), all of which varies in their modular structure, as well as the number of layers being considered for the image classification, competing to achieve superordinate performance vis-à-vis the recognition of daily objects. By encapsulating a unified modification to the structure of the last few layers of these models before the output, we also augmented their performance by inserting an extra fine-tuning step to the training process. Finally, amalgamating an ensemble model based on enhanced CNN models can heighten the diagnostic accuracy and the overall stability of the CAD system. All the CNN models we studied have been implemented in PyTorch and trained via Adam optimizer (Kingma and Ba, 2014).

#### Basic CNN Structure

A complete CNN architecture is composed of convolutional, pooling, and fully connected layers. As the core CNN building block, the convolutional layers work throughout the dataset to extract common patterns hidden within the local regions of the input image (Zeiler and Fergus, 2014). Here, outputs obtained from the process represent the combination of features extracted from the receptive field, with their relative position remaining unchanged. Other higher-level layers with weight vectors will then detect larger patterns from the original image to further process these outputs. Altogether, the shared weight vector provides a strong response on short snippets of data with specific patterns.

Pooling layers, typically placed after the convolutional layers, provide a method of non-linear down-sampling. They divide the output from the convolutional layers into disjoint regions, providing a single summary for each region to showcase the convolution characteristics. Before generating the classification result, one or more fully connected layers are typically placed at the very end of a CNN model. The fully connected layer structure develops a shallow multilayer perceptron, which purposes to integrate the local feature information previously extracted with categorical discrimination for classifying the input data.

#### Transfer Learning

Transfer learning, a process that stores the knowledge learned previously to be applied to a correlated task (Shin et al., 2016a), aims at leveraging previous learning to build accurate models for new specific tasks more efficiently (Pan and Yang, 2009).

In the computer vision field, deep, and complicated model structures are expensive to train because of the dataset size requirement and expensive hardware such as graphics processing units. Moreover, it can take weeks or longer to train a model from scratch; hence, using a pretrained model developed with internal parameters and well-trained feature extractors will often improve the model's overall performance to solve similar problems on relatively smaller datasets.

Accordingly, all CNN models used in our experiments are pretrained on the ImageNet dataset (Deng et al., 2009) to classify daily objects in 1,000 categories. Features learned from each layer have been extracted as the startup baseline for TB detection purposes. Not having to train from scratch, a lot of time will be saved. ImageNet is a state-of-the-art architecture used by several researchers (Jaeger et al., 2014; Hwang et al., 2016; Lakhani and Sundaram, 2017; Lopes and Valiati, 2017; Pasa et al., 2019) for pretraining the deep CNNs as it is already trained on 1.2 million everyday color images that consisted of 1,000 categories, before learning from the chest radiographs in this study. Anthimopoulos et al. (2016) work is well-documented to deploy in disease prediction based on CNN model, although it faces the problem of generalization and transferability. Similarly, Rajpurkar et al. (2018) tried with increasing number of layers in their work for increasing the accuracy, which is also not a foolproof approach, thus signifying the importance and necessity of developing the efficient hybridized, ensemble, and optimized model for better classification efficiency through faster convergence, which is yet to be addressed.

#### Structure Modification

Regardless of the integrated modules that execute the main feature extraction work, different CNN models vary in their general structures. For example, GoogLeNet Inception models do not have fully connected layer prior to generating the output whereas VGGNet and ResNet do. Similarly, ResNet models implement average pooling at the very last pooling layer, whereas VGGNet and GoogLeNet Inception models use max pooling. Simply put, to boost the performance of different deep CNN models and better utilize their internal parameters, a unified modification for the part that lies between the main modules and the output of original CNN architectures is needed (Parmaksizoglu and Alçi, 2011).

**Figure 2:**
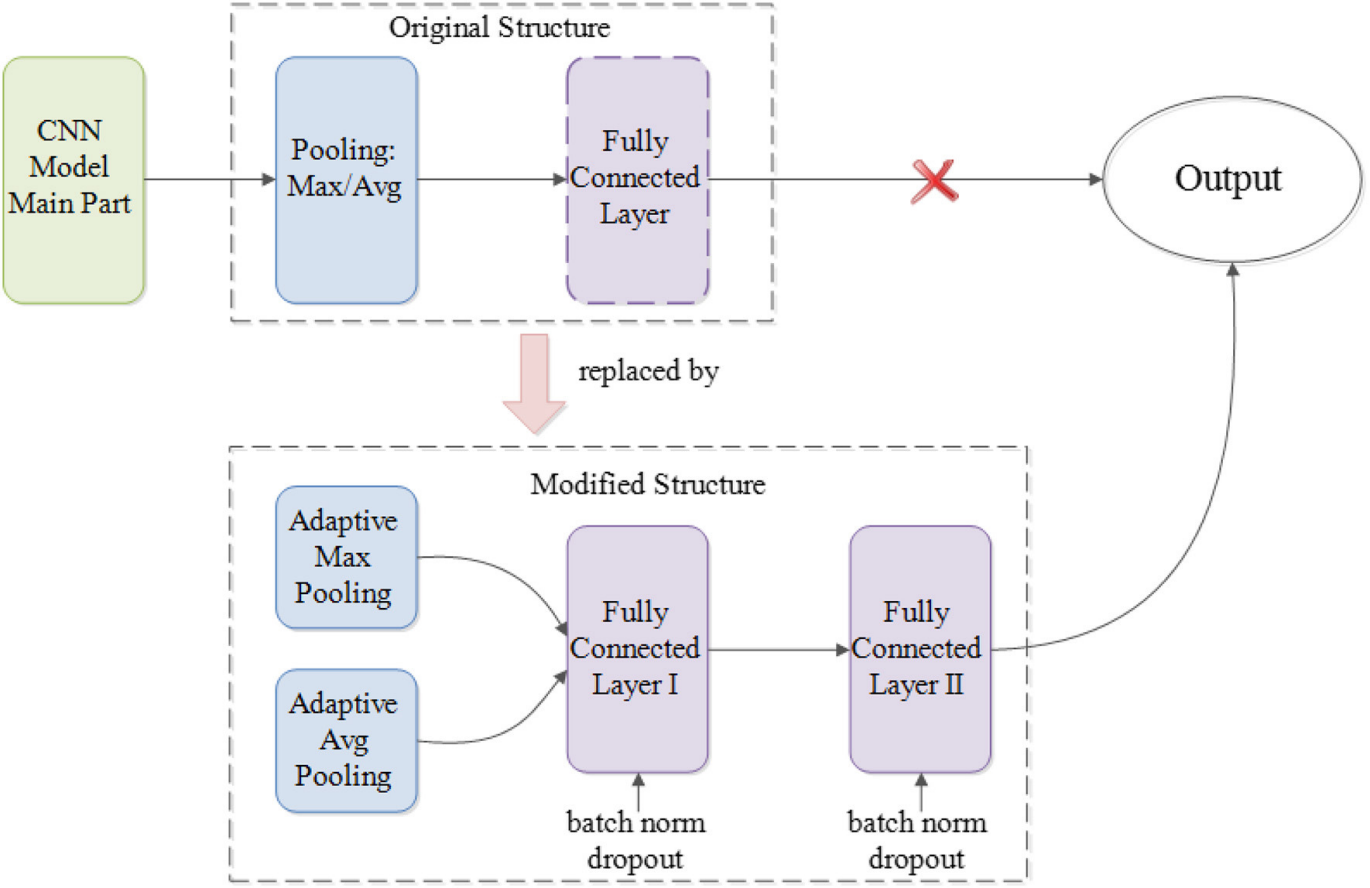
Modifications on CNN architecture.

As Figure 2 illustrated, such a modification entails having the very last pooling layer to be changed from the default settings of either max or average pooling into the parallel concatenation of adaptive max and average pooling. By integrating both maximized and averaged feature maps, it helps to accumulate more high-level information learned from the task dataset, ultimately generating more useful, and comprehensive details for future prediction. After adaptive pooling, two fully connected layers are added before the final output to generate a deep NN structure for better capturing and organization of the encapsulated information. Moreover, each fully connected layer has been embedded with both batch normalization and dropout functions. Whereas, batch normalization helps to eliminate the internal covariate shift of the activation values in feature maps so that the distribution of the activations remains the same during training, the dropout functions aim to prevent the overfitting problem caused by the overcomplicated structures.

#### Model Fine-Tuning via ABC

Mohd Aszemi and Dominic (2019) have suggested the use of genetic algorithm in tuning the hyperparameters in CNN. In another study, Serizawa and Fujita (2020) have proposed linearly decreasing weight particle swarm optimization for hyperparameters optimization of CNN. Parmaksizoglu and Alçi (2011) have proposed using the ABC algorithm for parameter tuning in CNN based edge detection in images. ABC, a metaheuristic algorithm (Karaboga, 2005), was inspired by the foraging behavior of bees; it has been abstracted into a mathematical model to solve multidimensional optimization problems (Karaboga and Basturk, 2007). Simply put, this algorithm represents solutions in multidimensional search space as food sources; more specifically, it maintains a population of three types of bees (scout, employed, and onlooker) to search for the best food source (Bullinaria and AlYahya, 2014). Horng (2017) proposed addressing the issue of properly fine-tuning parameters of deep belief networks by means of ABC algorithm. Hence, we have been encouraged to deploy the ABC optimization method for the tuning of parameters such as learning rate (LR), batch size, image subset, etc., for better classification accuracy. Certainly, there is a trade-off between exploitation and exploration while looking for solution (Hamed Mozaffari and Lee, 2020). Xu et al. (in press) have proposed the modified ABC (ABC-ISB) optimization algorithm for automatically training the parameters of feed-forward artificial neural networks. This clearly indicates the case of customization and variable behavior of the type of methods used in optimization. Generally, the biological data set has the inherent property of features variability where the behavior prediction is very tedious; hence, an optimized approach may be possibly the best solution.

In the early stage of collecting nectar, scout bees go out to find food sources by either exploring with the prior knowledge or via random search. Once the search task is over, the scout bee turns into an employed bee. Employed bees are mainly in charge of locating the nectar source and collecting nectar back to the hive. After that, based on specific requirements, they will proceed with the selection from continuing collecting nectar, dancing to attract more peers to help or give up the current food source and then change their roles either to scout or onlooker bees. The job of onlooker bees is to decide whether to participate in nectar collection based on the dance performed by employed bees.

Leveraging on the power of the ABC algorithm to obtain the globalized optimal solution, the ABC is applied in our study to fine-tune the fully connected layers of pretrained CNN models on CXR datasets to improve the resulting diagnostic accuracy. Essentially, the fine-tuning process may be regarded as searching for the appropriate parameters that could further minimize the total loss in the CNN model. Starting with randomly generated solutions, the ABC algorithm will iterate for better solutions by searching the nearby regions of the current best solution and abandoning those less desired solutions.

Initially, a solution vector that contains a specific number of possible solutions is created. Drawing on the previous training results, the first element of the solution vector is set with weights and biases taken from the trained CNN model. Multiplying the first solution vector with a random number between 0 and 1 generates the other elements nearby obtained weights and bias in the given space:

$$\begin{array}{l} sol\text{\_}vec=\left({w\left(t\right)}_{1},{w\left(t\right)}_{2},\ldots ,{w\left(t\right)}_{n}\right)\\ {w\left(0\right)}_{1}=\left(nn.W,\;nn.b\right)\\ {w\left(0\right)}_{i}=rand\left(0,\;1\right)\times {w\left(0\right)}_{1},\;i=2,3,\ldots ,n \end{array}$$

where *t* represents the total number of iterations needed during the whole fine-tuning process,

*n* denotes the number of possible solutions, and *nn*.*W* and *nn*.*b* are weights and biases of the CNN model, which are used as the baseline of other initialized solutions.

During optimization, not only will generalizing multiple solutions leverage the parameters from the trained model, but it will also prevent the model from iterating into local optimal points. Searching for the nearby solutions, *v*(*k*)_*i*_, will then be started based on the initialized vectors:

$$\begin{array}{l} gen\text{\_}vec=\left({v\left(t\right)}_{1},{v\left(t\right)}_{2},\ldots ,{v\left(t\right)}_{n}\right)\\ {v\left(k\right)}_{i}={w\left(k-1\right)}_{1}+\varphi_{i}\left({w\left(k-1\right)}_{i}-{w\left(k-1\right)}_{j}\right),\;i\neq j \end{array}$$

where *k* represents the *k*-th iteration of the optimization process, and φ is a random number that is uniformly distributed in the interval [0, 1].

Once the new solution nearby the initialized one is found, fitness value measuring the quality of solutions will be computed to compare between old and new solutions as per the following equation:

$$\begin{array}{l} fit\left({w\left(k\right)}_{i}\right)=\frac{1}{1+E\left({w\left(k\right)}_{i}\right)} \end{array}$$

where *E*(*w*(*k*)_*i*_), a non-negative value always, is the loss function of the CNN model, which is the target function that needs to be optimized. In our study, the loss function used is the cross-entropy loss (Zhang and Sabuncu, 2018):

$$\begin{array}{l} E\left({w\left(k\right)}_{i}\right)=-\frac{1}{n}\overset{n}{\underset{i=1}{\sum }}\left[y_{i}\ln\left({o\left(k\right)}_{i}\right)+\left(1-y_{i}\right)\ln\left(1-{o\left(k\right)}_{i}\right)\right] \end{array}$$

where *y*_*i*_ is the expected output of the *i*-th sample within the training batch, and *o*(*k*)_*i*_ is the actual output of this sample from the *k*-th iteration.

The selection of a better solution will then proceed based on the calculated probability of the fitness values:

$$\begin{array}{l} {p\left(k\right)}_{i}=\frac{fit\left({w\left(k\right)}_{i}\right)}{\sum_{i}fit\left({w\left(k\right)}_{i}\right)} \end{array}$$

Here, for each generated solution, the smaller the loss, the larger the fitness value, and there will be a greater probability to be selected as the final solution.

#### The Ensemble Model

Drawing on the idea of ensemble learning (Oza and Tumer, 2008), which integrates multiple classifiers and generates the final output based on results provided by the integrated classifiers to achieve better performance, an ensemble model is built. Classifiers used for ensemble purposes need to maintain sufficient diversity to capture different features from the same target. More generally, structuring an ensemble model requires two critical steps: (a) generate a distribution of simple models via the original data and (b) aggregate the distribution into a single model.

The underlying conceptualization is to learn data in a more unbiased way based on the knowledge learned by different classifiers. For instance, if a classifier learns a wrong feature pattern from the dataset, then a classification error for the new data having the similar feature may result; however, the tier-2 classifier of the ensemble model may learn things correctly by organizing the knowledge from all participating classifiers in an unbiased way to compensate for the individual classifier weaknesses, thereby generating the right classification result. This ability to provide a trade-off between bias and variances among base models as well as reducing the risk of overfitting makes the ensemble model superior to any other single structure (Kuncheva and Whitaker, 2003).

**Figure 3:**
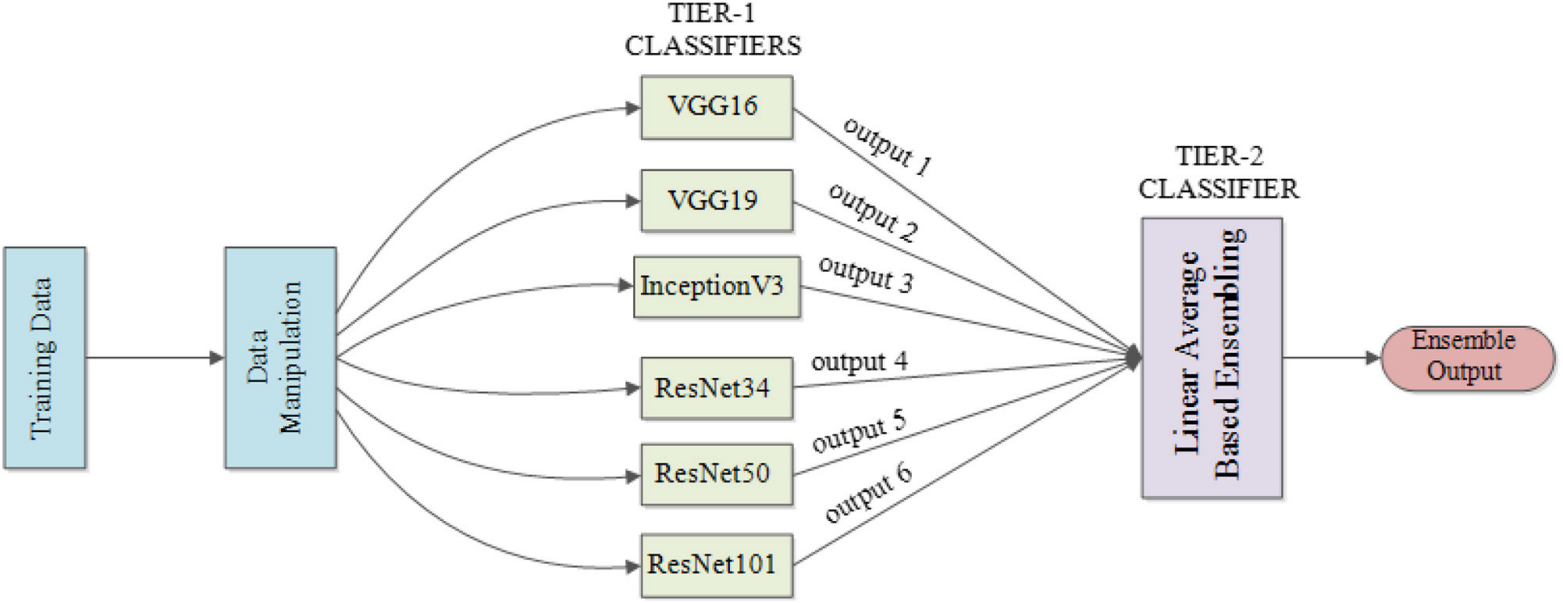
Proposed ensemble model structure used for TB diagnosis and localization.

Figure 3 illustrates the structure of our proposed ensemble model for TB diagnosis and localization. Here, the ensemble model is derived from computing the linear average of outputs from the component classifiers.

#### Localization Scheme

In a CNN model, the inner connection between the probability of classification result and the weights of the last few layers will shuffle to seek regions from the image that are correlated to the prediction results. As proposed by Zhou et al. (2016), the disease localization is established via class activation mapping.

**Figure 4:**
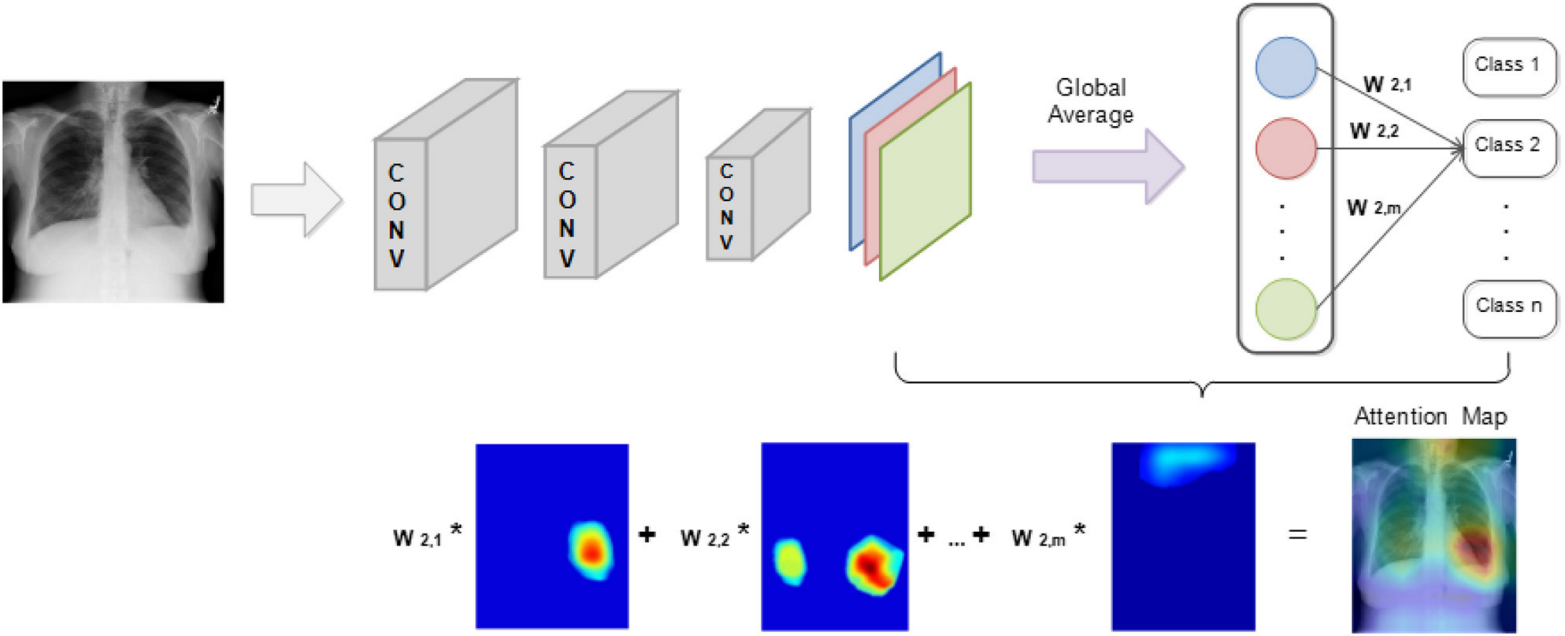
TB localization using class activation mapping.

Figure 4 illustrates localization in a CNN model that contains a global average pooling layer. Here, feature maps that are generated from the last convolutional layers are processed with global average pooling to generate a vector. The obtained vector will then be used to compute the weighted summation with the parameters of the fully connected layers to generate the output that can be used for classification. Hence, the weights from the last layer before the output can be projected back to the feature maps by connecting with the pooling layer to identify areas in which the model computed as exhibiting the important information.

The foregoing method fully utilized the pattern recognition and localization capabilities that exist in CNN models to attention map the target image. Importantly, by simply processing the internal parameters within a CNN, two different functions, image classification and object localization, on the same model can be successfully integrated. During the classification process, the generated attention map based on the input image identifies the regions that become the model's main predicting criteria.

#### Statistical Analysis

The performance parameters accuracy, specificity, recall, F1 score, and AUC were determined using 10-fold cross-validation. Further, different training-to-validation ratios of 7:3, 8:2, and 9:1 were used for statistically significant differences between the models using unpaired *t*-test for 95% confidence (*P* < 0.05) interval using R programming language. Generally, using 10-fold cross-validation violates the independence assumption due to resampling. McNemar test and 5 × 2 cross-validation (Dietterich, 2000) are commonly used to determine significant differences in the models; 10 × 10-fold cross-validation with the Nadeau and Bengio correction to the paired Student *t*-test was recommended by Witten and Hall (2011) to achieve good replicability. Latter strategy was used in this research. The overfitting problem is also addressed by the cross-validation.

## Results

### Experiment Settings

In our study, a binary classification of CXR images for lung abnormality diagnosis is performed for six different CNN models (VGG16, VGG19, Inception V3, ResNet34, ResNet50, and ResNet101). Again, both the Shenzhen Hospital CXR dataset and the NIH CXR dataset were evaluated. A further diagnosis and localization among seven TB-related manifestations are performed separately on the NIH CXR dataset. Ratio comparison for each disease identification task is also performed.

During experimentation, a certain amount of CXR images from the dataset is set aside for testing the final performance of a trained CNN model, whereas the rest of the dataset is split into training–validation sets at the patient level with the ratios of 9:1, 8:2, and 7:3, respectively. To achieve parallel comparisons of the trained CNN models on the same dataset, CXRs reserved for testing purposes in each dataset remain the same regardless of the variations in training–validation distribution.

Detailed separation of CXRs with different training–validation ratios within each dataset for the basic abnormality detection is given in Table 1.

**Table 1:** CXR separations for lung abnormality diagnosis.

**CXR dataset**		**Train/valid ratio = 9:1**		**Train/valid ratio = 8:2**		**Train/valid ratio = 7:3**		**9:1/8:2/7:3**
		**Training set**	**Valid set**	**Training set**	**Valid set**	**Training set**	**Valid set**	**Testing set**
Shenzhen hospital	Normal	270	30	240	60	210	90	6
CXR dataset	Abnormal with TB	285	35	250	70	225	95	16
Chest X-Ray8	Normal	29,250	3,250	26,000	6,500	22,750	9,750	1,831
dataset	Abnormal with TB	18,760	2,090	16,680	4,170	14,590	6,260	464

The original number of CXRs in the NIH CXR dataset used for specific TB manifestations and diagnosis is given in Table 2.

**Table 2:** Original CXR distribution of 7 TB-related manifestations in NIH CXR dataset.

**Consolidation**	**Effusion**	**Fibrosis**	**Infiltration**	**Mass**	**Nodule**	**Pleural thickening**
324	2,035	641	5,133	1,313	1,888	851

Clearly, the distribution of CXRs under each class presents a strongly biased trend. Models trained on this dataset perform a strong preference in their predictions; thus, data augmentation has been implemented to increase the number of images under the classes with fewer CXRs, thereby creating an evenly distributed data to eliminate the interference. Table 3 presents the distribution of CXRs after data augmentation. Note that images in the testing set have not been augmented in order to ensure the high quality of results via the testing of the models' performance on transfer learning.

**Table 3:** Augmented CXR distribution in NIH CXR dataset for TB-related manifestations diagnosis.

**TB manifestations**	**Train/valid ratio = 9:1**		**Train/valid ratio = 8:2**		**Train/valid ratio = 7:3**		**9:1/8:2/7:3**
	**Training set**	**Valid set**	**Training set**	**Valid set**	**Training set**	**Valid set**	**Testing set**
Consolidation	4,460	500	3,970	990	3,470	1,490	14
Effusion	4,500	500	4,000	1,000	3,500	1,500	85
Fibrosis	4,460	500	3,970	990	3,470	1,490	21
Infiltration	4,500	500	4,000	1,000	3,500	1,500	133
Mass	4,500	500	4,000	1,000	3,500	1,500	63
Nodule	4,500	500	4,000	1,000	3,500	1,500	88
Pleural thickening	4,500	500	3,980	1,000	3,480	1,500	21

### Disease Detection

#### Disease Identification via Single Models

Our initial experiment is to explore if our proposed modifications on deep CNN model structures with the application of ABC algorithm during model fine-tuning actually improve the accuracy of a single CNN model on detecting TB abnormality and identifying TB manifestations. For each CNN model classifying a specific dataset with different training/validation ratios, three stages of processing are included: (a) training with the original CNN architecture, (b) training with the modified CNN architecture, and (c) fine-tuning the trained modified CNN model via ABC.

At the beginning stage, all parameters within the CNN model that has the original architecture are fixed except for the last two layers, which are trained on the target CXR dataset for three epochs with an LR, 1e-3. As all CNN models used in our research are pretrained, features learned from previous layers must eventually transit from general to specific by the last two layers of the model that will have a direct influence on the final output. Only training the last two layers of a CNN model on new datasets for certain number of epochs in the beginning stages would reduce the time for the model to converge on a new task. Once the parameters of the CNN model have been trained on the last two layers, the entire model is then trained on the target datasets for 12 epochs with an LR, 1e-4. At the ending stage, the fully connected layer of each trained CNN model with modified architecture are fine-tuned via ABC algorithm to further improve the model's overall accuracy.

**Figure 5:**
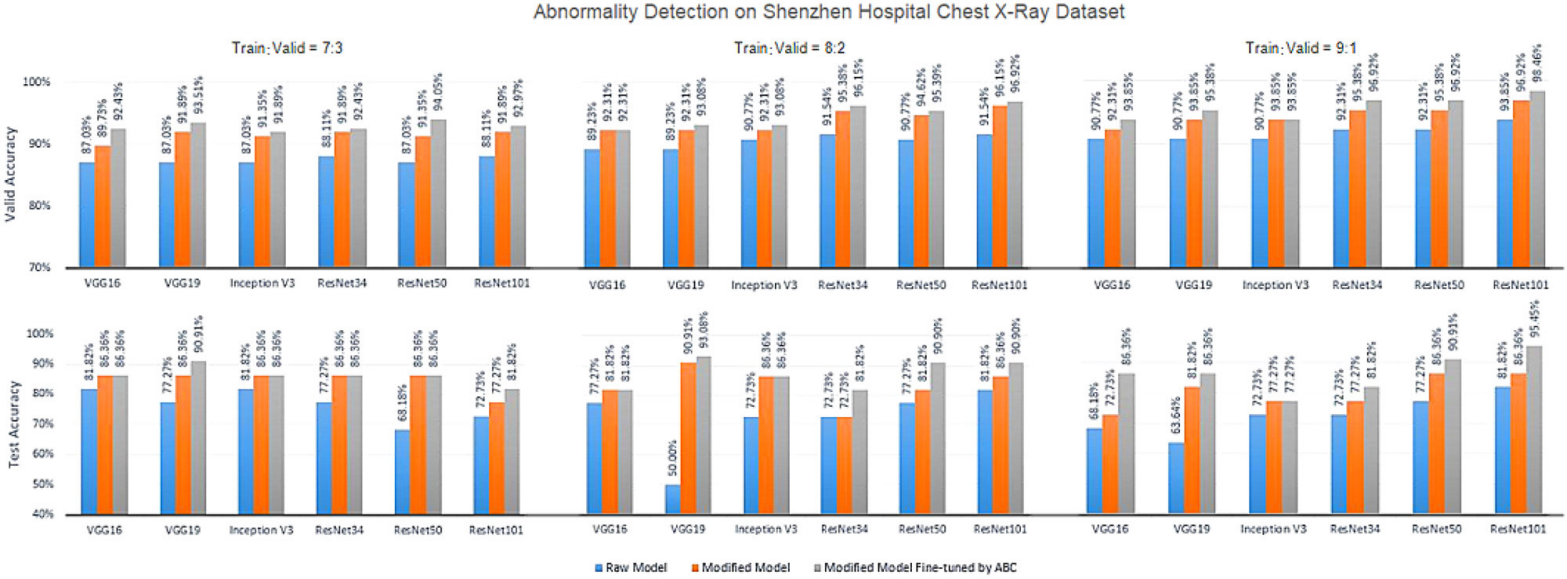
Diagnosis results on chest X-rays by the proposed ensemble model for detection of TB abnormality on Shenzhen Hospital Chest X-Ray dataset.

**Figure 6:**
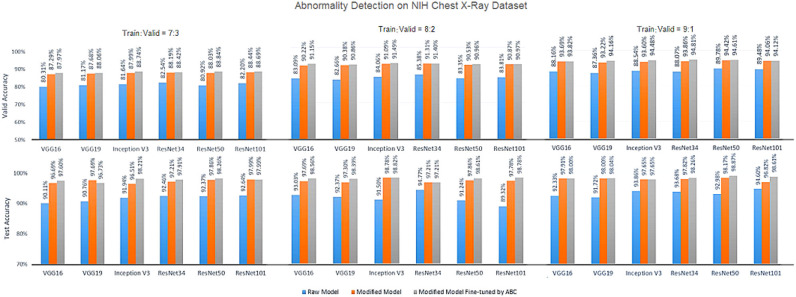
Diagnosis results on chest X-rays by the proposed ensemble model for detection of TB abnormality on NIH Chest X-Ray dataset.

**Figure 7:**
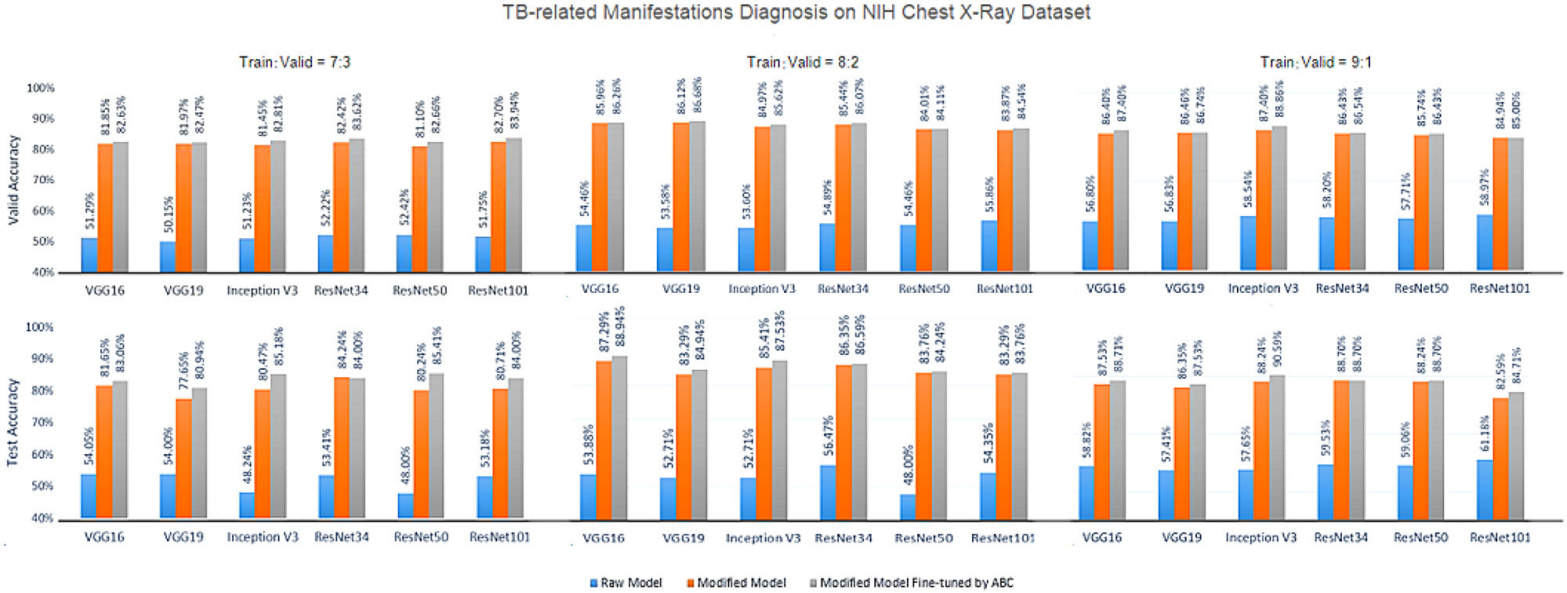
Diagnosis results on chest X-rays of the proposed ensemble model for detection of TB-relared manifestations on NIH Chest X-Ray dataset.

Figures 5–7 show the bar graphs to compare accuracy of the six deep CNN models for TB abnormality and manifestation detection on both the validation/testing datasets with training/validation ratios of 7:3, 8:2, and 9:1, respectively.

According to data portrayed by the bar charts, the modified CNN models generally present a significant improvement in the diagnostic accuracy on both validation/testing set of the two datasets with different training/validation ratios as compared to the original models. For the TB abnormality detection, the largest improvement in accuracy achieved is 40.91%, which has been observed in VGG19 on the Shenzhen Hospital CXR dataset with training/validation ratio 8:2. The improvement of diagnostic accuracy vis-à-vis the detection of specific TB-related manifestations is even more significant with an average improvement of >30% among all models on both the validation and testing set. With the extra fine-tuning step via ABC algorithm, the disease identification accuracy is further augmented. Although the enhancement is insignificant compared to that via structural modification, it did assist the CNN models to reach their best performance, generally achieving the highest accuracy across all models.

#### Disease Identification via the Ensemble Model

Following training and process improvement on individual CNN models, the linear average-based ensemble method is then implemented to further enhance the overall diagnostic performance.

Statistically, the following measures are used to evaluate the quality of the diagnostic performance, as well as the robustness of the model: accuracy, specificity, recall, F1 score, and AUC (the area under the receiver operating characteristic curve) score. High values for all of these evaluation metrics will be anticipated for an ideal classifier.

Whereas, specificity, recall, and F1 score are normally used to measure the performance on binary classification, accuracy pertains to measuring the proportion of correctly predicted cases among all input samples. Importantly, specificity reveals how many healthy people are correctly identified as not having TB-related manifestation. Recall aims at evaluating how many people who developed TB are correctly identified as having the TB-related manifestation. Finally, the statistical interpretation of AUC is that if choosing a case under a certain class randomly, what would be the probability that the selected class outranks other classes? This value is independent of the threshold set for the classification task because it only considers the rank of each prediction.

Tables 4, 5 highlight the comparison of the overall performance between the ensemble model and its component CNN models for the TB abnormality detection on the Shenzhen Hospital CXR dataset and the NIH CXR dataset, respectively. The performance of each component CNN model varies on each dataset with different training/validation ratios. Interestingly, as the number of images in the training set increases, the statistical measures of the ensemble model and its component CNN models improve likewise. Although the recall provided by the ensemble model is not the highest in most cases, overall, it presents better performance with the highest accuracy, specificity, F1 score, and AUC score. In our evaluation of the Shenzhen Hospital CXR dataset, the ensemble model provides the best diagnostic accuracy ranging from 94.59 to 98.46%, specificity from 95.57 to 100%, F1 score from 0.947 to 0.986, and AUC from 0.986 to 0.999 for the different training/validation ratios. For the NIH CXR dataset, the ensemble model provides the best diagnostic accuracy ranging from 89.56 to 95.49%, specificity from 96.69 to 98.50%, F1 score from 0.855 to 0.940, and AUC from 0.934 to 0.976 for the different training/validation ratios.

**Table 4:** Accuracy, specificity, recall, F1 score, and AUC scores comparison between single improved CNN models and the ensemble model for the detection of TB abnormality on Shenzhen Hospital CXR dataset when train/valid ratio equals 7:3, 8:2, and 9:1, respectively.

**Ratio**	**Model**	**Accuracy (%)**	**Specificity (%)**	**Recall (%)**	**F1 score**	**AUC**
7:3	VGG16	92.43	91.11	93.66	0.927	0.975
	VGG19	93.51	93.33	93.66	0.937	0.973
	Inception V3	91.89	90.00	93.66	0.922	0.963
	ResNet34	92.43	94.39	90.52	0.925	0.974
	ResNet50	94.05	94.39	93.66	0.942	0.964
	ResNet101	92.97	91.11	**94.71**	0.933	0.979
	Ensemble	**94.59**	**95.57**	93.66	**0.947**	**0.986**
8:2	VGG16	92.31	93.33	91.42	0.928	0.964
	VGG19	93.08	93.33	92.94	0.935	0.978
	Inception V3	93.08	91.78	94.33	0.936	0.973
	ResNet34	96.15	**96.78**	95.75	0.964	0.985
	ResNet50	95.39	93.33	97.12	0.958	0.986
	ResNet101	96.92	95.00	**98.67**	0.972	0.988
	Ensemble	**97.69**	**96.78**	**98.67**	**0.979**	**0.991**
9:1	VGG16	93.85	96.66	91.42	0.941	0.976
	VGG19	95.38	96.66	94.33	0.957	0.976
	Inception V3	93.85	90.00	97.12	0.944	0.980
	ResNet34	96.92	93.33	**100.00**	0.972	0.990
	ResNet50	96.92	**100.00**	94.33	0.971	0.991
	ResNet101	**98.46**	**100.00**	97.12	**0.986**	0.994
	Ensemble	**98.46**	**100.00**	97.12	**0.986**	**0.999**

*Statistics shown in bold font indicates the best results within each train/valid ratio group*.

**Table 5:** Accuracy, specificity, recall, F1 score, and AUC scores comparison between single improved CNN models and the ensemble model for the detection of TB abnormality on NIH CXR dataset when train/valid ratio equals 7:3, 8:2, and 9:1, respectively.

**Ratio**	**Model**	**Accuracy (%)**	**Specificity (%)**	**Recall (%)**	**F1 score**	**AUC**
7:3	VGG16	87.97	95.00	77.01	0.834	0.920
	VGG19	88.06	93.89	78.85	0.838	0.924
	Inception V3	88.74	96.42	76.94	0.842	0.924
	ResNet34	88.42	96.21	76.30	0.873	0.927
	ResNet50	88.84	96.08	77.58	0.845	0.928
	ResNet101	88.69	94.83	**79.18**	0.846	0.929
	Ensemble	**89.56**	**96.69**	78.52	**0.855**	**0.934**
8:2	VGG16	91.15	**97.13**	81.92	0.879	0.951
	VGG19	90.86	95.00	84.45	0.878	0.950
	Inception V3	91.49	95.52	85.24	0.887	0.952
	ResNet34	91.40	94.86	**86.00**	0.887	0.955
	ResNet50	90.96	96.31	82.73	0.877	0.948
	ResNet101	90.97	97.00	81.67	0.876	0.948
	Ensemble	**92.07**	97.00	84.45	**0.891**	**0.958**
9:1	VGG16	93.82	97.62	87.94	0.918	0.965
	VGG19	94.16	97.70	88.71	0.922	0.968
	Inception V3	94.48	97.03	90.62	0.928	0.974
	ResNet34	94.81	97.84	90.14	0.931	0.972
	ResNet50	94.61	97.84	89.60	0.929	0.970
	ResNet101	94.12	96.12	**91.12**	0.924	0.969
	Ensemble	**95.49**	**98.50**	90.91	**0.940**	**0.976**

*Statistics shown in bold font indicates the best results within each train/valid ratio group*.

Table 6 compares accuracy and AUC score between the ensemble model and its component CNN models for the diagnosis of the seven TB-related manifestations on the NIH CXR dataset with different training/validation ratios, respectively. In general, the ensemble model achieves the highest diagnostic accuracies among all TB-related manifestations. The only exception is for *consolidation*, when training/validation ratio is 8:2; here, the accuracy provided by the ensemble model is 82.93%, only 0.20% less than the highest one. More specifically, our ensemble model presents an outstanding performance on the diagnosis of *effusion, infiltration, mass*, and *nodule* with an overall accuracy of around 90% and AUC score of >0.988. The other three TB-related manifestations, including *consolidation, fibrosis*, and *pleural thickening*, have a relatively lower probability to be correctly detected, but still achieve an accuracy of >80%. Additionally, the consistency of providing both high AUC scores and prediction accuracies indicates that the model has an outstanding performance vis-à-vis the automatic ranking of patterns and the selection of threshold. Moreover, with an average AUC score of >0.975 on all TB-related manifestations, the ensemble model outranks the others by providing a robust performance, as well as a better probability of generating the prediction on the right disease.

**Table 6:** Accuracy and AUC score comparison between single improved CNN models and the ensemble model for the diagnosis of specific TB manifestations on NIH CXR dataset when train/valid ratio equals 7:3, 8:2, and 9:1, respectively.

**Ratio**	**Model**		**Consolidation**	**Effusion**	**Fibrosis**	**Infiltration**	**Mass**	**Nodule**	**Pleural thickening**
7:3	VGG16	Accuracy	78.46%	74.27%	83.29%	89.00%	81.13%	91.40%	80.87%
		AUC	0.959	0.979	0.963	0.978	0.984	0.981	0.965
	VGG19	Accuracy	81.95%	70.93%	79.73%	84.27%	91.07%	83.33%	83.73%
		AUC	0.962	0.980	0.964	0.977	0.983	0.982	0.965
	Inception V3	Accuracy	74.97%	80.20%	86.58%	82.07%	90.53%	83.47%	81.87%
		AUC	0.950	0.982	0.968	0.973	0.982	0.979	0.962
	ResNet34	Accuracy	79.19%	86.27%	81.93%	90.20%	89.47%	80.40%	77.27%
		AUC	0.967	0.980	0.971	0.975	0.986	0.979	0.966
	ResNet50	Accuracy	81.81%	84.53%	81.01%	85.13%	87.80%	77.80%	80.53%
		AUC	0.964	0.981	0.968	0.975	0.980	0.978	0.969
	ResNet101	Accuracy	74.83%	89.67%	81.74%	88.93%	87.80%	84.87%	79.47%
		AUC	0.954	0.982	0.967	0.976	0.985	0.981	0.972
	Ensemble	Accuracy	**84.32%**	**89.87%**	**87.25%**	**93.93%**	**93.47%**	**91.80%**	**86.73%**
		AUC	**0.975**	**0.990**	**0.979**	**0.988**	**0.993**	**0.993**	**0.982**
8:2	VGG16	Accuracy	79.39%	88.60%	84.85%	88.40%	84.20%	94.60%	83.70%
		AUC	0.957	0.987	0.978	0.981	0.987	0.989	0.973
	VGG19	Accuracy	82.02%	90.50%	81.82%	86.00%	89.10%	90.20%	87.00%
		AUC	0.973	0.986	0.978	0.978	0.987	0.989	0.972
	Inception V3	Accuracy	78.79%	88.20%	77.37%	88.50%	85.80%	94.40%	86.10%
		AUC	0.970	0.986	0.978	0.979	0.984	0.988	0.972
	ResNet34	Accuracy	81.31%	89.90%	80.20%	87.30%	90.20%	87.20%	86.30%
		AUC	0.962	0.987	0.971	0.983	0.988	0.988	0.974
	ResNet50	Accuracy	79.19%	88.00%	83.54%	86.40%	83.10%	87.20%	81.30%
		AUC	0.946	0.981	0.968	0.977	0.958	0.974	0.971
	ResNet101	Accuracy	**83.13%**	84.20%	81.92%	86.10%	90.00%	87.20%	86.20%
		AUC	0.970	0.984	0.976	0.979	0.985	0.987	0.977
	Ensemble	Accuracy	82.93%	**93.40%**	**86.36%**	**94.50%**	**92.50%**	**96.00%**	**88.00%**
		AUC	**0.979**	**0.993**	**0.984**	**0.990**	**0.993**	**0.996**	**0.985**
9:1	VGG16	Accuracy	79.20%	90.20%	86.40%	87.40%	92.40%	90.60%	85.60%
		AUC	0.960	0.985	0.972	0.984	0.988	0.990	0.963
	VGG19	Accuracy	79.20%	88.00%	88.40%	89.80%	87.80%	91.20%	84.20%
		AUC	0.945	0.984	0.970	0.987	0.987	0.988	0.974
	Inception V3	Accuracy	79.40%	89.00%	86.00%	95.20%	94.20%	91.20%	87.00%
		AUC	0.973	0.987	0.970	0.988	0.989	0.992	0.969
	ResNet34	Accuracy	80.40%	89.40%	86.20%	89.00%	83.80%	91.60%	84.60%
		AUC	0.964	0.987	0.970	0.983	0.985	0.989	0.969
	ResNet50	Accuracy	79.20%	86.40%	81.20%	91.20%	89.80%	91.40%	85.80%
		AUC	0.963	0.984	0.970	0.983	0.983	0.985	0.974
	ResNet101	Accuracy	79.80%	87.40%	86.00%	86.20%	87.40%	87.00%	81.20%
		AUC	0.948	0.982	0.966	0.975	0.982	0.983	0.967
	Ensemble	Accuracy	**81.20%**	**93.60%**	**89.80%**	**96.40%**	**95.60%**	**95.20%**	**88.00%**
		AUC	**0.976**	**0.991**	**0.985**	**0.994**	**0.992**	**0.996**	**0.979**

*Statistics shown in bold font indicates the best results within each train/valid ratio group*.

#### Disease Localization

**Figure 8:**
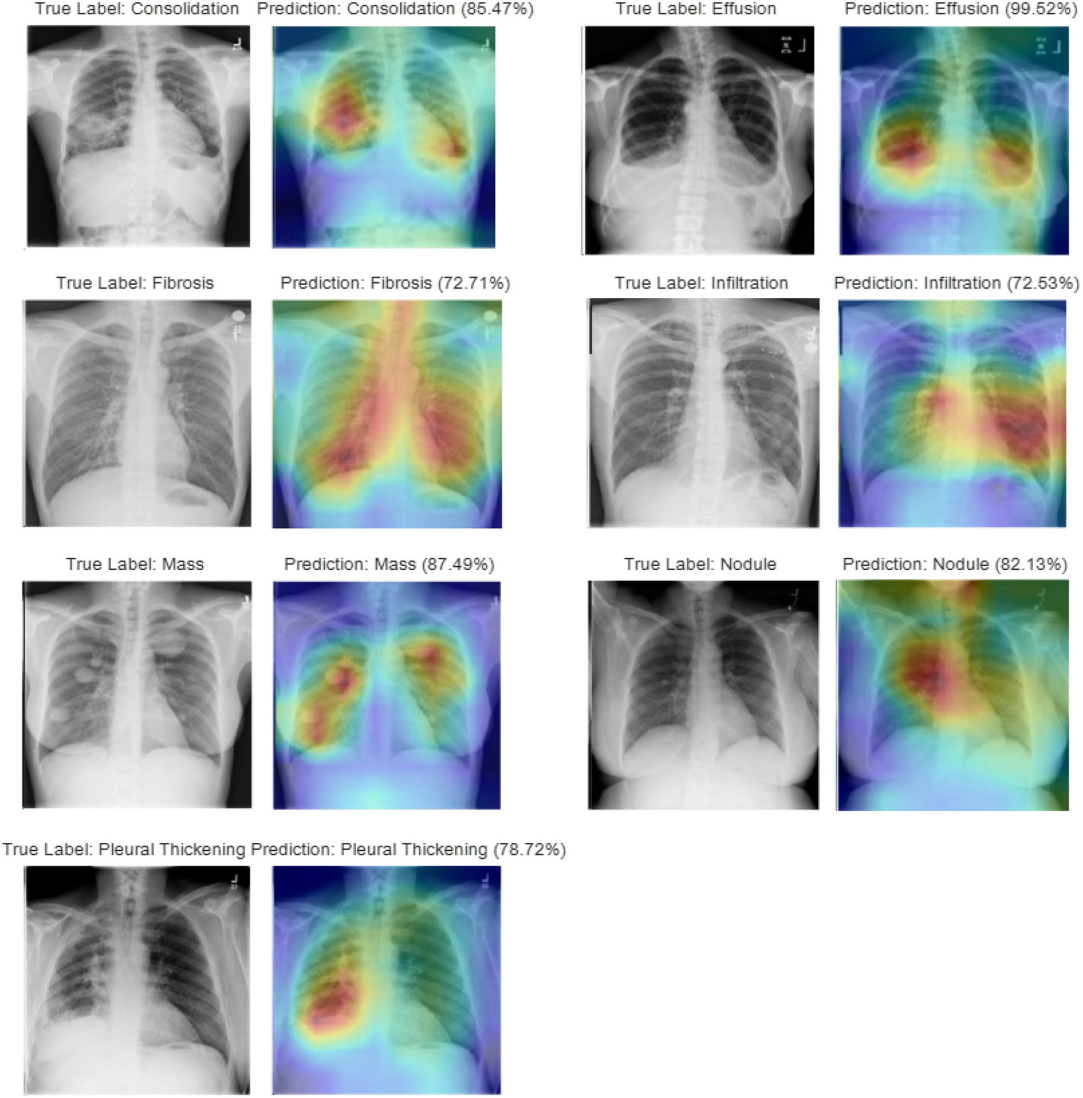
Visualization of disease localization on test images for each TB-related disease by class activation mapping and attention maps.

In studying disease localization, the class activation mapping is implemented on the ensemble model to offer visualization on the location of the detected TB-related manifestations. Figure 8 shows some exemplary localization results on various test cases for each TB-related manifestation. Areas highlighted in red and yellow, which have been used to assist doctors and radiologists for more in-depth examination during the clinical practice, are the suspicious candidate regions.

Multiple physicians and radiologists have affirmed that our diagnostic and localization results are consistent with the clinical TB manifestation signs. Intuitively, we see that the attention map generated by our ensemble model, which relates to the predicted TB manifestations, covers >97% of the diseased area. As an example, during the diagnosis of *consolidation*, a disease caused by the disappearance or reduction of the gas in the alveolar cavity, not only can our model locate the suspicious regions that contain the compressed lung tissue, but the model also can distinguish it from other diseases with similar appearance (i.e., *infiltration*) or caused by similar reasons (i.e., *effusion*). Similarly, during the localization of lung *nodules*, even for a small focal density that covers an extremely small region (≤ 3 cm in diameter) of the entire CXR, our model will still provide satisfying performance by spotlighting the specified area containing the suspected symptoms. For diseases (i.e., *effusion, mass*) that may appear at multiple places within the lung areas, the generated attention map can mark all regions with the detected manifestations.

## Discussion

Our experiments investigated both the detection of lung abnormality and the diagnosis of specific TB manifestations among seven TB-related lung diseases (*consolidation, effusion, fibrosis, infiltration, mass, nodule*, and *pleural thickening*) from CXR images via deep CNN models. Different training/validation ratios (7:3, 8:2, and 9:1) have been used to find the optimal model.

For detecting lung abnormality, apart from accuracy and AUC score, the most commonly used performance measures include specificity, recall, and F1 score, all of which have also been used to provide an overall analysis on both the diagnostic accuracy and the robustness of the models. Among all deep CNN models evaluated, our proposed ensemble model achieves the best accuracy of 98.46%, a specificity of 100%, an F1 score of 0.986, and AUC of 0.999 with a training/validation ratio of 9:1, as well as the best recall of 98.76% with a training/validation ratio of 8:2 on the Shenzhen Hospital CXR dataset. For the same dataset, Lakhani and Sundaram (2017) achieved the best AUC of 0.99, whereas Pasa et al. (2019) achieved the best AUC of 0.925. Our results not only showcase a significant improvement on the AUC score, but also provide a more comprehensive estimation measures, viz., accuracy, specificity, F1 score, and recall.

Table 7 shows a comparison of the state-of-the-art results on the Shenzhen TB CXR dataset with our ensemble model. Results show that the proposed method gives superior results compared to the state-of-the-art.

**Table 7:** Comparison between state-of-the-art and the best results of our ensemble model for the detection of TB abnormality on Shenzhen Hospital CXR dataset.

**State-of-the-art literature**	**Accuracy (%)**	**Specificity (%)**	**Recall (%)**	**F1 score**	**AUC**
Jaeger et al. (2014)	84				0.900
Hwang et al. (2016)	83.7				0.926
Lopes and Valiati (2017)	84.7				0.926
Lakhani and Sundaram (2017)					0.990
Pasa et al. (2019)					0.925
Rajaraman and Antani (2020)	94.1	95.7	92.6	94.1%	0.990
Proposed method	**98.46**	**100.00**	**97.12**	**0.986**	**0.999**

*Statistics shown in bold font indicates the best results*.

Altogether, our analysis shows how the model performs in classifying the input CXRs; importantly, the model correctly identifies the healthy cases as no abnormality vs. those sick people as having lung abnormalities. The model is robust with the capability of distinguishing the input case to a target class. As for the lung abnormality detection performed on the NIH CXR dataset, our ensemble model achieves the best accuracy of 95.49%, a specificity of 98.50%, an F1 score of 0.940, and AUC of 0.976 with training/validation ratio of 9:1. The highest recall achieved by the ensemble model is 90.91%, that is, 0.21% less than the best recall provided by ResNet101.

To the best of our knowledge, research on the binary classification for detecting lung abnormality vis-à-vis the NIH CXR dataset is lacking. In this area, our work provides a baseline via a state-of-the-art algorithm. In diagnosing the specific TB manifestations among seven TB-related lung diseases from CXR images in the NIH CXR dataset, our proposed ensemble model achieves an accuracy range of 81.20 to 84.32% and AUC in the range of 0.975–0.979 for the diagnosis of *consolidation*, an accuracy range of 89.87–93.60%, and AUC in the 0.990–0.993 range for the diagnosis of *effusion*, an accuracy range of 86.36–89.80% and AUC in the range of 0.979–0.985 for the diagnosis of *fibrosis*, an accuracy range of 93.33–96.40% and AUC in the range of 0.988–0.994 for diagnosing *infiltration*, an accuracy range of 92.50–95.60% and AUC in the range of 0.992–0.993 for diagnosing *mass*, an accuracy range from 91.80–96.00% and AUC in the range of 0.993–0.996 for diagnosing *nodule*, and an accuracy range of 86.73–88.00% and AUC in the range of 0.979–0.985 for diagnosing *pleural thickening*. Rajpurkar et al. (2018) reported AUC score of 0.7901 on *consolidation*, 0.8638 on *effusion*, 0.8047 on *fibrosis*, 0.7345 on *infiltration*, 0.8676 on *mass*, 0.7802 on *nodule*, and 0.8062 on *pleural thickening* on the NIH CXR dataset. Apparently, our results significantly outperform this baseline, whereas our analysis provides a comprehensive diagnostic prediction measures on the detection of TB-related manifestations on CXRs from the NIH CXR dataset as compared to those of Rajpurkar et al. (2018). Unlike most of the research work that only focuses on AUC scores during the diagnosis of multiple diseases, we provide the diagnostic accuracy, recall, specificity, F1 score, and AUC for each disease to better analyze the overall performance of our model.

Class activation mapping is implemented to localize suspicious regions that contain the detected TB-related manifestations. The capability to highlight the suspicious diseased area via an attention map provides an efficient interpretation of the diagnostic results generated by CNN. Clinically verified by radiologists, our model provides an impressive performance on distinguishing one TB-related disease from other diseases with similar appearance or caused by similar reasons. Finally, the model has the ability to generate the attention map that covers multiple areas with the suspected disease (e.g., *effusion, mass*) and even with only tiny little abnormality that takes only 1/10,000 of the entire CXR image (e.g., *nodule*). Liu et al. (2019) proposed an end-to-end CNN-based locating model for pulmonary TB diagnosis in radiographs on datasets provided by Huiying Medical Technology (Beijing) Co., Ltd. With labels marked by cross-validation of expert doctors, and the seconds one was selected and provided by Henan Provincial Chest Hospital. However, this study did not provide localization of TB-related manifestations. Luo et al. (2019) proposed a RetinaNet based CNN pipeline to automatically detect, localize, and subclassify suspected TB on chest radiographs under its three main pulmonary presentations (cavitary, infiltrate, and miliary) on a prior pneumonia dataset, the RSNA Pneumonia Detection Challenge. Xue et al. (2018) proposed a method based on CNNs for locating TB in CXRs to classify the superpixels generated from the lung area consisting of four major components: lung ROI extraction, superpixel segmentation, multiscale patch generation/labeling, and patch classification. Their method was tested on a publicly available TB CXR dataset, which contains 336 TB images showing various manifestations of TB. The TB regions in the images were marked by radiologists. To date, our work is among the first that has presented localization of TB-related manifestations via attention maps on the NIH CXR dataset.

## Conclusions

In summary, a unified modification to the deep CNN model structure and fine-tuning of the model via the ABC algorithm during the model training process have been proposed to accurately predict the diagnosis of TB-related diseases and localization of specific disease manifestation. Multiple deep CNN models (VGG16, VGG19, Inception V3, ResNet34, ResNet50, ResNet101) varying in the structure of modules as well as the number of layers had been applied to test our proposed methodology. A linear average–based ensemble model composed of those improved CNN models is implemented and applied to further improve the overall performance.

Our results show that with the superimposition of the improvement steps, the overall performance of deep CNN models keeps getting better. Among the three steps, structure modifications generate the largest increment on the prediction accuracy for single CNN models. The fine-tuning step applying the ABC algorithm helps to improve performance slightly. By assembling the individual CNN models, the classification accuracy of CXRs is further improved. Moreover, each model presents an unstable and unpredictable performance on different datasets and for different classification tasks; with the employment of the ensemble models, the classification accuracy reaches the highest, and the robustness has been greatly improved. Even for the disease localization task, our proposed ensemble model can present satisfying result by accurately providing an attention map to spotlight regions of the suspicious diseased lung area. Both quantitative and qualitative results demonstrate that our methodology offers an outstanding performance compare to other state-of-the-art algorithms.

## Data Availability Statement

Publicly available datasets were analyzed in this study. This data can be found here: https://lhncbc.nlm.nih.gov/publication/pub9931; https://cloud.google.com/healthcare/docs/resources/public-datasets/nih-chest.

## Author Contributions

RG did data curation, formal analysis, investigation, formulated methodology, found resources, developed the software, performed visualization, and wrote original draft. KP conceptualized the project, did formal analysis, did investigation, devised the methodology, administered the project, found resources, supervised the project, validated the results, reviewed, and edited the manuscript. CJ conceptualized the project, did investigation, devised the methodology, administered the project, supervised the project, reviewed, and edited the manuscript. All authors contributed to the article and approved the submitted version.

## Conflict of Interest

The authors declare that the research was conducted in the absence of any commercial or financial relationships that could be construed as a potential conflict of interest.
